# Akt Is S-Palmitoylated: A New Layer of Regulation for Akt

**DOI:** 10.3389/fcell.2021.626404

**Published:** 2021-02-15

**Authors:** Matías Blaustein, Estefanía Piegari, Camila Martínez Calejman, Antonella Vila, Analía Amante, María Victoria Manese, Ari Zeida, Laurence Abrami, Mariela Veggetti, David A. Guertin, F. Gisou van der Goot, María Martha Corvi, Alejandro Colman-Lerner

**Affiliations:** ^1^Departamento de Fisiología, Biología Molecular y Celular (DFBMC), Facultad de Ciencias Exactas y Naturales (FCEN), Universidad de Buenos Aires (UBA), Buenos Aires, Argentina; ^2^Instituto de Fisiología, Biología Molecular y Neurociencias (IFIBYNE), Consejo Nacional de Investigaciones Científicas y Técnicas (CONICET)-UBA, Buenos Aires, Argentina; ^3^Instituto de Biociencias, Biotecnología y Biología Traslacional (iB3), Universidad de Buenos Aires, Buenos Aires, Argentina; ^4^Program in Molecular Medicine, University of Massachusetts Medical School, Worcester, MA, United States; ^5^Laboratorio de bioquímica y biología celular de parásitos, Instituto Tecnológico de Chascomús (IIB-INTECH), Universidad Nacional de San Martín (UNSAM) – CONICET, Chascomús, Argentina; ^6^Departamento de Bioquímica and Centro de Investigaciones Biomédicas (CEINBIO), Facultad de Medicina, Universidad de la República, Montevideo, Uruguay; ^7^Global Health Institute, École Polytechnique Fédérale de Lausanne (EPFL), Lausanne, Switzerland; ^8^Department of Molecular, Cell and Cancer Biology, University of Massachusetts Medical School, Worcester, MA, United States; ^9^Lei Weibo Institute for Rare Diseases, University of Massachusetts Medical School, Worcester, MA, United States

**Keywords:** Akt, S-palmitoylation, cell signaling, subcellular localization, Golgi, lysosomes, autophagy, cell differentiation

## Abstract

The protein kinase Akt/PKB participates in a great variety of processes, including translation, cell proliferation and survival, as well as malignant transformation and viral infection. In the last few years, novel Akt posttranslational modifications have been found. However, how these modification patterns affect Akt subcellular localization, target specificity and, in general, function is not thoroughly understood. Here, we postulate and experimentally demonstrate by acyl-biotin exchange (ABE) assay and ^3^H-palmitate metabolic labeling that Akt is S-palmitoylated, a modification related to protein sorting throughout subcellular membranes. Mutating cysteine 344 into serine blocked Akt S-palmitoylation and diminished its phosphorylation at two key sites, T308 and T450. Particularly, we show that palmitoylation-deficient Akt increases its recruitment to cytoplasmic structures that colocalize with lysosomes, a process stimulated during autophagy. Finally, we found that cysteine 344 in Akt1 is important for proper its function, since Akt1-C344S was unable to support adipocyte cell differentiation *in vitro.* These results add an unexpected new layer to the already complex Akt molecular code, improving our understanding of cell decision-making mechanisms such as cell survival, differentiation and death.

## Introduction

To process external and internal information, cells have evolved a multiplicity of signaling systems. At least 15% of the vertebrate’s genome protein coding capability is linked to receptors, components of signaling systems and transcription regulatory proteins and their dysfunction is associated with several pathologies (Koonin et al., 2004). In the case of cancer, changes in the function of signaling systems are so important that they are utilized to define and classify different types of tumors (Hanahan and Weinberg, 2011).

Signaling pathways often transduce information through phosphorylation events mediated by protein kinases. Akt (also known as protein kinase B or PKB), a serine/threonine protein kinase member of the AGC family, is vastly studied as a model of how signaling proteins transduce and process extracellular information into cell decisions and fates (Staal, 1987; Blaustein, 2018). Akt plays a central role in growth, proliferation, glucose uptake, metabolism, angiogenesis, protein translation, cell survival as well as in viral infection (Diehl and Schaal, 2013; Manning and Toker, 2017). Not surprisingly, a variety of human cancers exhibit dysregulated Akt activity and several mouse models with activated Akt develop cancer (Altomare and Testa, 2005; Toker and Yoeli-Lerner, 2006; Riggio et al., 2012). Thus, it is not surprising that different clinical trials are underway to test the efficacy of a variety of inhibitors of the Akt pathway as anticancer and antiviral treatments (Dunn et al., 2009; Okuzumi et al., 2009).

Akt contains an N-terminal pleckstrin homology (PH) domain that interacts with phosphatidylinositol (3,4,5)-triphosphate (PIP3) (Andjelkovic et al., 1997; Franke et al., 1997), a central kinase domain and a C-terminal domain that contains an hydrophobic motif (HM) with homology to other AGC kinases (Alessi et al., 1996). There are three isoforms of Akt in mammals (Akt1, Akt2, and Akt3), each transcribed from a separate gene (Jones et al., 1991a,b; Konishi et al., 1995; Brodbeck et al., 1999). Traditional activation of all Akt isoforms is triggered when class I phosphatidylinositol-3-kinase (PI3K), which converts plasma membrane (PM) associated phosphatidylinositol-4,5-bisphosphate (PIP2) to PIP3, is recruited by activated receptor tyrosine kinases (Geering et al., 2007). Cytosolic Akt then relocalizes to the PM by binding to PIP3, allowing its phosphorylation at T308 (T309 and T305 in Akt2 and AktT3, respectively) by Phosphoinositide-Dependent Kinase-1 (PDK1) and at S473 (S474 and S472 in Akt2 and Akt3, respectively) by the mammalian target of rapamycin complex 2 (mTORC2) (Alessi et al., 1997; Sarbassov et al., 2005). Interestingly, recent findings show that phosphatidylinositol-3,4-bisphophate (PI34P2), also produced by PI3K, selectively recruits Akt2 at both the PM and early endosomes, whereas PIP3 specifically binds to Akt1 and Akt3 at the PM (Liu et al., 2018).

Different groups have shown that Akt can be phosphorylated in several residues other than T308 and S473 (Risso et al., 2015; Manning and Toker, 2017). In addition, Akt undergoes other posttranslational modifications (PTMs): O-glycosylation, ubiquitination, acetylation, oxidation and SUMOylation (Park et al., 2005; Yang et al., 2009; Sundaresan et al., 2011; Wani et al., 2011a,b; Risso et al., 2013), highlighting that regulation of Akt activity is exceptionally complex (Risso et al., 2015; Manning and Toker, 2017). Recently, it was also demonstrated that Akt is proline-hydroxylated (Guo et al., 2016) and methylated (Yoshioka et al., 2016; Guo et al., 2019; Wang et al., 2019). If all the reported Akt PTMs were independent from each other, the number of potential states of Akt would be larger than one hundred million (a number that is even higher if the three Akt paralogues and their splicing isoforms are taking into account). In addition, Akt has been associated with a number of subcellular destinations: activated Akt acts on multiple targets located in the PM, cytosol and nucleus. Remarkably, Akt was also found (presumably on the cytosolic side) in other subcellular compartments such as mitochondria, endoplasmic reticulum (ER) and lysosomes (Hosoi et al., 2007; Santi and Lee, 2010; Arias et al., 2015). Consistently, several Akt substrates and functions have been reported to be associated with newly described Akt subcellular destinations (Mounir et al., 2011; Wang et al., 2012; Blaustein et al., 2013; Matsuda-Lennikov et al., 2014; Arias et al., 2015).

Undoubtedly, the information contained in the repertoire of Akt PTMs, through what we call the still undeciphered “Akt molecular code,” might contribute to relocalize Akt to a particular subcellular compartment, target a specific pool of substrates and trigger a specific response. Here, we studied whether Akt is modified by S-palmitoylation, a reversible thioester linkage of palmitate to cysteine residues catalyzed by internal membrane-bound palmitoyl acyltransferases (PATs) (Salaun et al., 2010; Martin et al., 2012). S-palmitoylation is common in several proteins localized to the ER, lysosomes and the PM, including some upstream and downstream signaling components of the Akt pathway, such as Ras (Magee et al., 1987) and eNOS (Haynes et al., 2000). hAkt1 has a predicted S-palmitoylation site at C344, which is also present in hAkt2 and 3 and is conserved in mouse, rat, *C. elegans* and even in the yeast *S. cerevisiae* closest homologue, Sch9. Here, we show that Akt is indeed S-palmitoylated and that Akt1-C344S mutation affects Akt phosphorylation and localization patterns. Palmitoylation-deficient Akt increases the recruitment of this kinase to cytoplasmic structures that colocalize with lysosomes, particularly in response to autophagic stimuli. Importantly, Akt1-C344S was unable to support adipocyte cell differentiation *in vitro.*

## Materials and Methods

### Cell Culture, Treatments, and Materials

Cell lines were grown and transfected according to the manufacturer’s instructions using standard procedures (Blaustein et al., 2009). HEK293T and HeLa Kyoto cells were grown in high glucose (4.5 g/L glucose) Dulbecco’s modified Eagle’s medium (DMEM) supplemented with 10% fetal bovine serum (FBS) and penicillin/streptomycin (100 units/ml and 100 μg/ml respectively, Thermo Fisher Scientific) in a 37°C humidified incubator containing 5% CO2. HEK293T medium was also supplemented with 110 mg/L of sodium pyruvate. Cells were plated (3 × 10^6^ cells per 100 mm plates, 2 × 10^5^ cells per well for 6 well plates and 1 × 10^4^ cells per well for 96 well glass-bottom imaging plates) and grown for 24 h before transfection and for another 24 h before treatment for the specified times. All cell lines were transfected with FuGENE6 (Roche) or Lipofectamine 2000 (Thermo Fisher Scientific) according to the manufacturer’s instructions. Unless otherwise stated, all experiments were performed in DMEM supplemented with 10% FBS. IGF-1 and 2-BP were obtained from Sigma-Aldrich. Lysotracker Red (Thermo Fisher Scientific) was a kind gift from Dr. Daniel Hochbaum.

The inducible KO Akt1/2/3 preadipocyte cell line was generated and differentiated as previously described (Cederquist et al., 2017; Sanchez-Gurmaches et al., 2018). To generate this cell line, primary brown adipocyte precursors (bAPC) cells were isolated from P1 neonates, immortalized with pBabe-SV40 Large T antigen and were cultured in high glucose-DMEM with 10% fetal calf serum (Hyclone) under 5% CO_2_. To induce Akt1 and Akt2 deletion, Akt1^*fl*/*fl*^ Akt2^*fl*/*fl*^ (both alleles of Akt1 and Akt2 flanked by loxP sites) Akt3 KO cells containing the estrogen regulated Cre UBC-Cre ERT2 construct were treated on 2 consecutive days with 1 mM 4-hydroxy tamoxifen and on the 3rd day the media was changed to regular media to allow protein turnover. KO Akt1/2/3 pre-adipocytes were infected with pBABE-pura-HA retroviruses into which WT Akt1, Akt1-E17K or Akt1-C344S coding sequences were cloned. For *in vitro* differentiation, pre-adipocytes cells were seeded at 4 × 10*^4^* cells/ml and allowed to reach confluence over 3 days in medium containing 20 nM insulin, 1nM T3 (differentiation medium). On day 4, cells were induced to differentiate with 20 nM insulin, 1nM T3, 0.125 mM indomethacin, 2 μg/mL dexamethasone and 0.5 mM 3-isobutyl-1-methylxanthine. Two days later the induction medium was replaced with fresh differentiation medium and changed every two days until day 10.

### Plasmids

pCMV6-HA-Akt1/2 and mutants derived from them have been previously described (Ramaswamy et al., 1999; Yang et al., 2009). Wild type Akt1/2 and mutants were subcloned into pEYFP-N1, pECFP-N1 and pECFP-C1 (Clontech) as well as in pBABE-puro-HA (for infection of Adipocytes) by PCR using appropriated primers as already described (Blaustein et al., 2013). C344S mutagenesis was performed by PCR. TGN38-FRB-CFP and PDK1-GFP were a kind gift from Dr. V. Laketa. pCS-memb-mCherry was a kind gift from Pablo Mammi and Anabella Srebrow.

### ABE Assay

HEK293T cells in 100-mm plates were transfected with either WT or mutant versions of pCMV6-HA-Akt1/2 and pAkt1-YFP. After 48 h, 90% confluent cells were washed with PBS and lysed at 4°C in 2 mL lysis buffer (50 mM Tris-HCl pH 7.4, 150 mM NaCl and 5 mM EDTA containing EDTA-free protease inhibitor (Roche)). Two plates from each construct were pooled (2 mg total protein/4 ml lysis buffer) and subjected to ABE assay as previously described (Wan et al., 2007), with the following modification: the cell lysate containing 10 mM NEM was sonicated 15 s on/off for 10 periods and then the concentration of NEM was adjusted to 2 mM for overnight treatment. The rest of the procedure was performed as described by Wang and coworkers (Wan et al., 2007). Briefly, after overnight treatment to block free thiol groups, NEM was removed by sequential chloroform-methanol precipitation. Half of each sample was then treated with hydroxylamine and biotin-HPDP to exchange thiol-bound fatty acids for biotin. Finally, proteins were precipitated to remove free biotin. An aliquot (20% of this sample) was taken after the biotinylation step to be used as the input (I) for each sample. The remainder of the samples (PD) were pulled down using NeutrAvidin-agarose resin (Thermo Scientific), and then eluted with 5 mM DTT. The total amount of each fraction, corresponding to eluates and inputs (80 and 20%, respectively) was run in parallel in a 10% SDS-PAGE for subsequent transfer onto a PVDF membrane, immunoblotted and quantified using ImageJ to obtain a PD/I value. An important control omits the hydroxylamine treatment step in the other half of each sample, to reveal either inappropriate biotinylation or non-specific streptavidin–agarose binding. These minus- hydroxylamine (-HA) sample proteins are also non-specifically purified into the experimental +HA sample and thus represent a source of false-positive signal in the assay. Thus, the fraction of S-palmitoylated Akt in each sample was obtained by subtracting non-specific pull down (PD/I) (-HA) from specific PD/I (+HA) values.

### ^3^H-Palmitate Metabolic Labeling

For ^3^H-palmitate metabolic labeling, HeLa Kyoto cells were starved for 1 h in minimal media and then incubated for 2 h in minimal media supplemented with 200 μCi/mL ^3^H-palmitic acid [9,10-^3^H(N)] (American Radiolabeled Chemicals ART 0129). Cells were then lysed and lysates were immunoprecipitated with antibodies against HA tag. After immunoprecipitation, proteins were eluted, and samples were divided for Western blotting (one fourth) and gel fixation (three fourths). Gels were exposed to fixing solution (25% isopropanol and 10% acetic acid in H2O), followed by a 30 min incubation with signal enhancer Amplify NAMP100 (Amersham) and gel drying. Dried gels were exposed to Hyperfilm MP (Amersham) film for 10-day to 6 week. In some cases, 1 M hydroxylamine (pH 7.4) was added and samples were incubated at room temperature for 5 min before loading on SDS/PAGE. Western blots were done in parallel to show the efficiency of the immunoprecipitation.

### Western Blot Analysis

Protein extract preparation and western blot analysis were performed as previously described (Blaustein et al., 2009). Primary antibodies were all from Cell Signaling: Akt (#9272), phospho-Akt (T308, #4056, S473, #4060 and T450, #9267), GSK-3β (#9315) and phospho-GSK-3β (S9, #9336), except for HA (MMS-101R, Covance) and GFP (11814460001, Roche). Fluorescence from secondary antibodies IRDye 680RD and IRDye 800RD (LI-COR Biosciences) was captured using an Odyssey Imaging System (LI-COR Biosciences). To quantify the bands obtained we used an Image Studio Lite (LI-COR Biosciences) based analysis.

### Molecular Dynamics Simulations

A complete protein structural determination for Akt1 is still not available. MD simulations for wild type Akt1, the S-palmitoylated form in C344 and the phosphorylated form in T308 (pT308) were performed using as initial model the *Homo sapiens* structure (PDBID: 4ekk) (Lin et al., 2012) where only the kinase and regulatory domains, i.e., residues from 144 to 477 were included as in previous studies (Lu et al., 2015; Mou et al., 2017). The *apo* form was generated ruling out the phosphoaminophosphonic acid-adenylate ester and the Mn^2+^ cation from the active site. C344 was modeled in the thiolate form as it is required for S-palmitoylation. The Amber ff14SB force field was used for protein residues (including pT308) (Maier et al., 2015), the palmitoyl group parameters were extracted from the GAFFlipid force field (Dickson et al., 2012) and the C344-palmitoyl thioester bond was constructed. The systems were placed into a truncated octahedral box of TIP3P water and after a standard minimization and equilibration protocol, 200 ns long production molecular dynamics (MD) were performed as described (Hugo et al., 2017) using the Amber package (Case et al., 2005). Trajectories visualization and drawings were performed with the Visual Molecular Dynamics (VMD) program (Humphrey et al., 1996). Eigenvector centrality measurements were performed as described (Negre et al., 2018). Eigenvector centrality considers the number of connections of a given node and its relevance in terms of information flow. It is defined as the weighted sum of the centralities of all nodes that are connected to it by an edge and it is particularly relevant to characterize allosteric mechanisms.

### Cell Imaging and Image Analysis

For fluorescent reporter assays, cells were plated either into 96-well (Matrical) or 8-well (Nunc^®^ Lab-Tek^®^ II) coverglass imaging plates. After transfection and treatment, cells were fixed with 4% paraformaldehyde in PBS for 5 min at room temperature, washed twice with PBS, permeabilized for 5 min with 0.2% Triton X-100 in PBS and stained with DAPI or Hoechst (Thermo Fisher Scientific). Lysotracker Red (Thermo Fisher Scientific) was added to live cells 1h before fixation. Wide-field images were captured on an Olympus IX-81 fluorescence microscope equipped with a 63X PlanApo oil immersion objective (N.A. = 1.4), a motorized XYZ stage and a Coolsnap HQ2 CCD camera (Photometrix, Tucson AZ). Acquisition was carried out with MetaMorph 7.5 software (Universal Imaging Corporation, Downingtown, PA). MetaMorph was also used to control the built-in motorized XYZ-motor and cube changer. Image processing and analysis were performed using the open source softwares ImageJ and CellProfiler. Cell segmentation was performed with the open source software CellProfiler. First, background subtraction and illumination heterogeneities corrections were performed with the module “Apply threshold” to nuclei, Akt, and Golgi images. Nuclei segmentation was performed with the module “IdentifyPrimaryObjects” on DAPI/Hoechst images while entire cells were segmented with the module “IdentifySecondaryObjects” on the Akt images. Clumped objects were distinguished by shape and cell lines between them were drawn taking into account their shape as well. In all cases we used global strategies and the Otsu method. After measuring cell shape and fluorescence intensity, cells that were too big, too small or did not express enough fluorescent reporter were filtered and discarded. Golgi was segmented using the enhance operation on “EnhanceOrSuppressFeatures” module and selecting the feature type as “Speckles.” Then “IdentifyPrimaryObjects” module was applied with strategies per object and “RobustBackground” thresholding method. Clumped objects were distinguished by intensity and cell lines between them were drawn taking into account their intensity as well. Then, the individual Golgi structures segmented were grouped with the “RelateObjects” module. At the end, the cytoplasm object was segmented using the “IdentifyTertiaryObjects” module by subtracting the organelles objects corresponding to each cell. For each object, we measured mean and total signal as well as the area. Akt recruitment to each subcellular compartment was measured as its mean intensity in the region. Each value was normalized by the corresponding Akt cytosolic mean intensity to correct for Akt expression levels. Data analysis and graphs were fulfilled using RStudio and Matlab. At least 100 cells were analyzed for each experiment. Colocalization of Akt-C344S and lysosomes (or DNA as a control) was determined calculating the Pearson’s correlation coefficient with the ImageJ Coloc2 plugin. The mean +/- standard deviation from different regions was computed. Akt recruitment to PM was measured as the average ratio between PM (maximum) and cytosolic (minimum) Akt signals in Region of Interest (ROI) from different cells, using ImageJ.

### Gene Expression Analysis

RNA isolation, retro-transcription and quantitative RT-PCR were performed using standard procedures. Total RNA was isolated from cells using Qiazol reagent (Invitrogen) and RNeasy kit (Invitrogen). Equal amounts of RNA (2 μg) were retro-transcribed to cDNA using a high-capacity cDNA reverse transcription kit (#4368813, Applied Biosystems). Quantitative RT-PCR was performed in 10 μL reactions using a StepOnePlus real-time PCR machine from Applied Biosystems using 2X SYBR Green PCR master mix (#B21203, Biomake.com) according to manufacturer instructions. Standard and melting curves were run in every plate for every gene to ensure efficiency and specificity of the reaction. Tbp expression was used as a housekeeping gene in all RT-PCR experiments. Primer information is listed in the table below.

Primer sequences for quantitative RT-PCR analysis:

**Table:** 

**Gene**	**Forward primer (5′-3′)**	**Reverse primer (5′-3′)**
Tbp	GAAGCTGCGGTACAATTCCAG	CCCCTTGTACCCTTCACCAAT
Pparγ2	TCAGCTCTGTGGACCTCTCC	ACCCTTGCATCCTTCACAAG
Acly	CTCACACGGAAGCTCCATAA	ACGCCCTCATAGACACCATC
Acaca	GGAGATGTACGCTGACCGAGAA	ACCCGACGCATGGTTTTCA
Fasn	GCTGCGGAAACTTCAGGAAAT	AGAGACGTGTCACTCCTGGACTT

### Oil Red O (ORO Staining)

Cells were fixed with 4% paraformaldehyde, washed with distilled water and treated with 100% propylene glycol twice for 5 min followed by a 15 min incubation with oil red O solution. Then cells were washed with 85% propylene glycol for 3 min and rinsed twice in distilled water.

### Statistical Analysis

Comparisons were performed using one-tailed Student’s *t* test and one-way analysis of variance (ANOVA), combined with Tukey’s test. Except otherwise stated, data are represented as the mean +/- standard error of the mean (SEM) from three independent experiments.

## Results

### Akt Undergoes S-Palmitoylation

**FIGURE 1:**
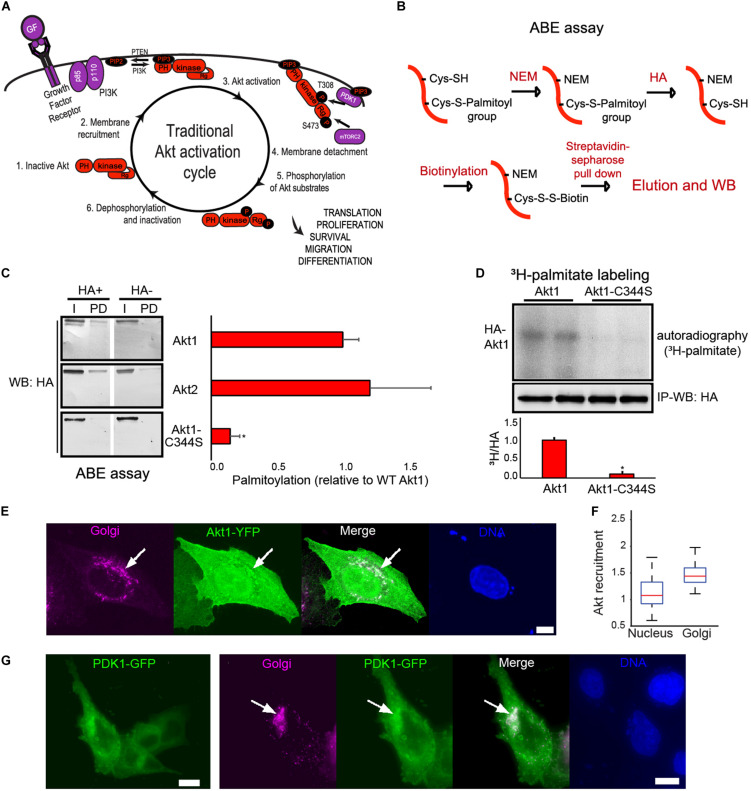
Akt is S-palmitoylated. **(A)** A traditional perspective of the Akt activation cycle assumes that PI3K, which converts PM associated PIP2 to PIP3 in response to Growth Factors (GF) and other stimuli, leads to PIP3- mediated recruitment of cytosolic Akt to PM. The conformational change elicited by Akt PH domain binding to PIP3 allows phosphorylation of Akt by PDK1 and mTORC2. As a result, activated Akt concertedly regulates a great variety of targets and functions in the cytosol and the nucleus. **(B)** Scheme of the ABE assay. It consists of three steps: Blocking free thiols with *N*-ethylmaleimide (NEM), conversion of thioester-linked palmitoyl moieties into thiols with a hydroxylamine (HA) treatment and biotinylation with biotin–HPDP. **(C)** Bar plots show the fraction of palmitoylated HA-Akt relative to HA-Akt1 (mean +/- SEM) in transiently transfected in HEK293T cells (similar results were obtained using HA or YFP tags), as determined using the ABE assay. An aliquot of each sample was taken before Pull-down (PD) and was used to measure the input (I). In HA- samples, the hydroxylamine treatment step was omitted. The fraction of palmitoylated Akt in each sample was obtained by subtracting non-specific pull down (PD/I) (-HA) from specific PD/I (+HA) values. The values were expressed as relative to WT Akt1 palmitoylation. *p < 0.05 compared to WT Akt1 (Student’s *t*-test). **(D)** HeLa Kyoto cells were either transfected with plasmids coding for WT HA-Akt1 or HA-Akt1-C344S and labeled with ^3^H-Palmitate, followed by immunoprecipitation of HA-Akt1. Radioactive signal and western blots are shown. ^3^H-palmitate to HA-Akt1 (^3^H/HA) ratios were plotted as relative to WT Akt1 palmitoylation. *p < 0.05 compared to WT Akt1 (Student’s *t*-test). **(E)** HeLa Kyoto cells were co-transfected with a plasmid coding for Akt1-YFP (green) and a plasmid coding for a Golgi fluorescent marker (TGN38-FRB-CFP, magenta). DAPI was used to reveal cell nuclei (blue); scale bar, 5 μm. **(F)** Box plot shows Akt recruitment to Golgi (and nucleus, as a reference) in HeLa Kyoto cells (*n* > 100). Akt recruitment to each subcellular compartment was measured as its mean intensity in the region. Each value was normalized by the corresponding Akt cytosolic mean intensity to correct for Akt expression levels. Median appears in red, the first and third quartiles in blue and minimum/maximum as error bars. **(G)** HeLa Kyoto cells were co-transfected with a plasmid coding for PDK1-GFP (green) and a plasmid coding for a trans-Golgi fluorescent marker (TGN38-FRB-CFP, magenta). DAPI was used to reveal cell nuclei (blue); scale bar, 10 μm.

It is usually assumed that inactive, cytosol residing Akt is recruited to the PM in response to extracellular stimuli resulting in its phosphorylation and detachment. This fully activated version of Akt orchestrates the regulation of a great variety of targets and functions in the cytosol and the nucleus in a concerted fashion (Figure 1A). However, recent data shows that Akt activation and relocalization mechanisms, including its recruitment to internal membranes, are more complex and, so far, not fully understood (Risso et al., 2015; Guo et al., 2016; Manning and Toker, 2017; Liu et al., 2018). Some proteins linked to the Akt signaling pathway, such as Ras (Magee et al., 1987) and eNOS (Haynes et al., 2000), shuttle between ER, Golgi, lysosomes and PM using S-palmitoylation at Golgi and/or ER membranes as a reversible anchor. Because Akt itself localizes to internal membranes, we speculated that the pool of Akt proteins residing at these membranes could be also regulated by this PTM.

To test the hypothesis that Akt is S-palmitoylated, we first used the CSS-Palm prediction tool (Ren et al., 2008). Indeed, CSS-Palm indicated that hAkt1 has an S-palmitoylation motif centered on cysteine C344 (C345 and C341 in hAkt2 and 3, respectively). Notably, this motif is conserved in mice, rats, worms and in the closest Akt homolog present in *S. cerevisiae*, Sch9 (Supplementary Table 1). Interestingly, the described SNPs around C344 in human Akt1 (such as variant rs56289559) are either synonymic or maintain the S-palmitoylation consensus according to the Variation Viewer (NCBI) and the Genome Aggregation Database (gnomAD) (Karczewski et al., 2020), suggesting strong selection against losing this consensus.

Next, we assessed if Akt was S-palmitoylated using an acyl-biotin exchange (ABE) assay (Wan et al., 2007). In this assay (Figure 1B), the palmitoyl group is exchanged by a sulfhydryl-reactive biotin derivative (biotinamido hexyl-pyridyl dithio propionamide, Biotin-HPDP), and then streptavidin is used to pull-down the labeled proteins, which are finally detected, for example, by western blot. In this way, we detected that Akt1 and Akt2 were indeed S-palmitoylated in extracts of unstimulated HEK293T cells transfected with tagged forms of these kinases (Figure 1C). Palmitoylation was reduced to background levels when we transfected the mutant Akt1-C344S, indicating that cysteine 344 was the target of this PTM (Figure 1C). To verify bona-fide Akt palmitoylation, we performed *in vivo* metabolic labeling of a different cell line, HeLa Kyoto, using ^3^H-palmitate, followed by immunoprecipitation of Akt1. After separation in an SDS-PAGE gel, a radioactive band corresponding to Akt could be clearly detected. As with the ABE assay, this band was absent when using cells transfected with Akt1-C344S (Figure 1D).

The above results indicated the presence of a fraction of palmitoylated Akt in basal, unstimulated HEK293T and HeLa Kyoto cells. Inactive Akt has been suggested to be a cytosolic protein. The highly hydrophobic nature of the palmitate group made the scenario of a soluble cytosolic palmitoylated Akt unlikely. One possibility is that the palmitoylated Akt molecules we detected were attached to internal cell membranes in unstimulated cells. The fact that several PATs are Golgi-membrane enzymes (Salaun et al., 2010; Martin et al., 2012) caused us to examine if Akt could be found in this location. Indeed, we observed a significant colocalization of Akt-YFP with a Golgi marker in a large proportion of unstimulated HeLa Kyoto cells (Figures 1E,F), suggesting that a subpopulation of Akt molecules might be palmitoylated in these membranes in basal cell-culture conditions. Moreover, we found that PDK1-GFP, the Akt activating kinase, also colocalized with the Golgi marker (Figure 1G), placing it in the appropriate location to phosphorylate Akt in this subcellular structure. All together, these results are consistent with the possibility that in unstimulated cells the Golgi subpopulation of Akt molecules could be palmitoylated by resident PATs, and eventually, activated by PDK1.

**FIGURE 2:**
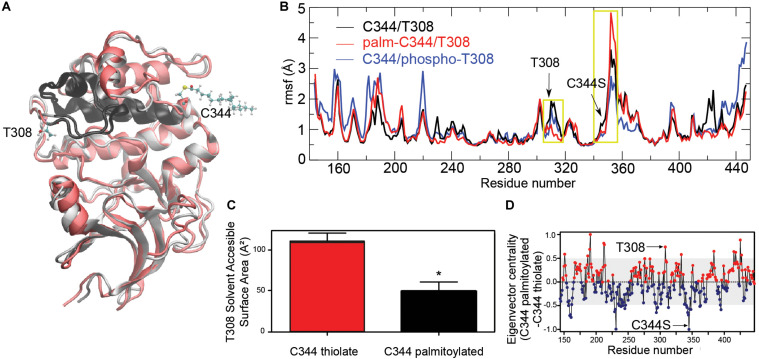
Effect of S-palmitoylation on Akt 3D structure. **(A)** Alignment of unmodified (gray) and S-palmitoylated (red) representative structures of apo-Akt1 model. C344, T308 and palmitoyl group are depicted in balls and cylinders for the S-palmitoylated protein. Protein backbone connecting T308 and C344 is highlighted in black. **(B)** Root mean square fluctuation (rmsf, Å) per residue for unmodified Akt1 (black), palmitoylated-C344 Akt1 (red) or phospho-T308 Akt1 (blue). Yellow boxes highlight T308 and C344 areas. **(C)** Comparison of solvent accessible surface area (SASA, Å^2^) of T308 obtained from unmodified (black) and S-palmitoylated (red) MD simulations. *p < 0.01 compared to C344 thiolate (Student’s *t*-test). **(D)** Eigenvector centrality measurement of mutual information for characterization of T308-C344 structural connection.

We next wondered how S-palmitoylation of Akt might affect its 3D structure. To answer this, we performed molecular dynamics simulations of Akt1 with different PTMs (Figure 2A). In our simulations, S-palmitoylation at C344 (Figure 2B, red line) generated changes in conformational freedom with respect to the unpalmitoylated original form (black line), but only in a few areas of the simulated kinase. Notably, a strongly affected region (large difference between black and red) was the area near T308, the key threonine whose phosphorylation by PDK1 is indispensable for Akt1 activation (Figures 2B,C). Reciprocally, simulation of T308 phosphorylation (Figure 2B, blue line) led to a change in the conformational freedom of the area near C344. These results suggested that these two regions (the neighborhood of C344 and T308) are structurally linked. In support of this notion, eigenvector centrality measurement of mutual information, a recently developed strategy to characterize the range of correlations that underlie allosteric processes (Negre et al., 2018), also suggested the presence of a structural allosteric effect connecting changes at C344 and T308 (Figure 2D).

Taken together, our simulation data raised the possibility that Akt S-palmitoylation might not only regulate its membrane association but also its activation state. This is consistent with some reports that indicate that sometimes S-palmitoylation influences other PTMs, including phosphorylation, therefore affecting protein activity (Salaun et al., 2010).

### Akt1-C344 Mutant Displays Altered Phosphorylation Patterns

**FIGURE 3:**
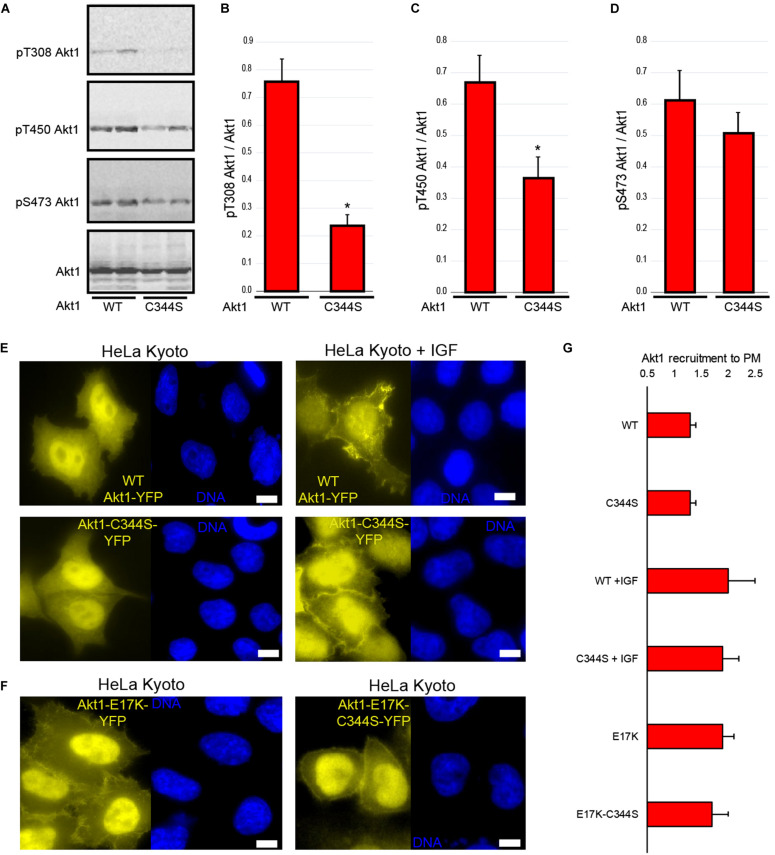
C344S mutation affects Akt1 phosphorylation. **(A–D)** HEK293T cells were transfected with WT or C344S Akt1-YFP. Protein extracts were analyzed by western blot using the indicated antibodies (duplicates are shown). Data in the plots corresponds to the ratio (mean +/- SEM) of phosphorylated to total YFP-tagged Akt. *p < 0.05 compared to WT in each case (Student’s *t*-test). **(E–G)** HeLa Kyoto cells were transfected with WT Akt1-YFP or Akt1-C344S-YFP **(E)**, or Akt1-E17K-YFP or Akt1-E17K-C344S-YFP **(F)** (yellow). Cells were serum-starved and treated with 100 ng/mL IGF-1 for 5 min or left untreated. DAPI was used to reveal cell nuclei (blue); scale bar, 5 μm. Akt recruitment to PM **(G)** was measured as the average ratio between PM (maximum) and cytosolic (minimum) Akt signals in Region of Interest (ROI) from different cells, using ImageJ. No significant differences were found between WT and C344S in each case (Student’s *t*-test).

Our molecular dynamics simulations suggested that Akt S-palmitoylation at internal membranes could be influencing Akt activation and particularly PDK1-dependent Akt phosphorylation at T308. Interestingly, we found that phosphorylation at T308 was decreased 3-fold in the Akt1-C344S mutant (Figures 3A,B), suggesting that the non-palmitoylated Akt1 could be less active. In addition, phosphorylation at T450 was also reduced in Akt1-C344S relative to WT Akt1 (Figures 3A,C), while no significant differences were observed regarding phosphorylation of S473 (Figures 3A,D).

Given that Akt1-C344S lacks the anchoring function provided by the palmitoyl group, we also wondered if it has a diminished capacity to be recruited to the PM, as it has been shown for other proteins (Sato et al., 2009). To test that, we transfected cells with either WT Akt1-YFP or Akt1-C344S-YFP, stimulated them with IGF-1 and measured Akt1 translocation to the PM (Figures 3E,G). We found no significant differences in PM recruitment between WT Akt1 and the C344S mutant. To complement this approach, we evaluated the ability of C344S mutation to affect PM recruitment of the oncogenic Akt1-E17K mutant (Figure 3F). The Akt-E17K mutant has been identified in human cancer patients and exhibits clear PM recruitment even in the absence of stimulus (Carpten et al., 2007). This is because the E17K mutation is in the PH domain and it has been shown to change the phospholipid specificity increasing binding to PIP2 instead of PIP3 (Landgraf et al., 2008). Again, we found that introduction of the C344S mutation did not alter PM recruitment (Figure 3G). These two experiments suggest that PM recruitment might be palmitoylation independent. However, more detailed studies are needed to rule out a role of this PTM in PM localization.

### Palmitoylation-Deficient Akt Increases Its Recruitment to Cytoplasmic Structures That Colocalize With Lysosomes

**FIGURE 4:**
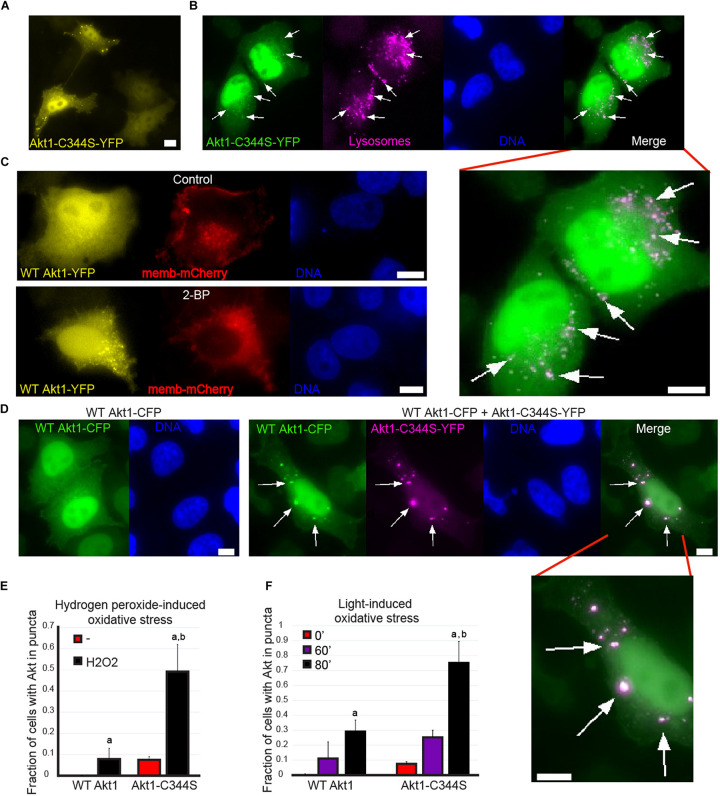
A link between Akt S-palmitoylation, lysosome recruitment and the response to oxidative stress. **(A)** An example of untreated cells displaying Akt1-C344S-YFP in cytoplasmic puncta; scale bar, 5 μm. **(B)** HeLa Kyoto cells were transfected with a plasmid coding for Akt1-C344S-YFP (green) and live stained with Lysotracker Red to observe lysosomes (magenta). DAPI was used to reveal cell nuclei (blue); scale bar, 5 μm. The Pearson’s correlation coefficient was calculated in different ROI (marked with arrows) obtaining an average of 0.71 +/– 0.12 between Akt1-C344S-YFP and Lysotracker Red (as a control, a correlation coefficient of 0.17 +/– 0.28 was obtained when comparing Akt1-C344S-YFP and DNA). **(C)** HeLa Kyoto cells were co-transfected with plasmids coding for a PM fluorescent marker (pCS-memb-mCherry, red) and WT Akt1-YFP (yellow). 48 h later, cells were treated with 100 μM of the palmitoylation inhibitor 2-BP or DMSO (vehicle) for 1 h and fixed. A significant increase in the proportion of cells displaying WT Ak1-YFP in puncta (one at least) was found when we treated them with 2-BP (from 1% in control to 30% in cells treated with 2-BP, *p* < 0.05 according to Student’s *t*-test). PM localization of pCS-memb-mCherry depends on the palmitoylable N-terminal sequence of Lyn kinase and therefore it was used as a positive control of the effect of 2-BP. DAPI was used to reveal cell nuclei (blue); scale bar, 5 μm. **(D)** HeLa Kyoto cells were transfected with a plasmid coding for Akt1-C344S-YFP (magenta) and/or a plasmid coding for WT Akt1-CFP (green). After 48 h, cells were fixed. Left: normal WT Akt1-CFP localization. Right: Localization of WT Akt1-CFP in cells with Akt1-C344S-YFP puncta. DAPI was used to reveal cell nuclei (blue); scale bar, 5 μm. **(E)** HeLa Kyoto cells were transfected with a plasmid coding for either WT or Akt1-C344S-YFP. Forty-eight hours later, cells were incubated for 3 h in the presence or absence of 10 mM hydrogen peroxide, fixed, and cells were scored as having or not YFP signal in puncta. Data in the plots corresponds to the fraction of cells containing YFP in puncta (mean +/- SEM). Letters indicate significant differences (ANOVA). a. *p* < 0.05 compared to untreated cells expressing the same version of Akt. b. *p* < 0.05 compared to WT Akt1-YFP cells under the same treatment. **(F)** HeLa Kyoto cells were transfected with a plasmid coding for either WT or Akt1-C344S-YFP. Live cells were imaged every 20 min to deliberately induce photo-damage. At every time point, we performed a Z Series with 3 z steps of 1 μm for 3 different wavelengths (RFP, exposure: 0.2 s; YFP, exposure: 0.5 s; CFP, exposure: 0.1 s) and cells were scored as having or not YFP signal in puncta. Data in the plots corresponds to the fraction of cells containing YFP in puncta (mean +/- SEM). Letters indicate significant differences (Student’s *t*-test): a. *p* < 0.05 compared to the same cells at *t* = 0’. b. *p* < 0.05 compared to WT Akt1-YFP cells at *t* = 80’.

A small but significant fraction of cells (between 5 and 10%) transfected with the S-palmitoylation mutant Akt1-C344S-YFP accumulated fluorescence in the form of cytoplasmic puncta (Figure 4A). This was not observed in WT Ak1-YFP overexpressing cells (Figures 3E, 4B), suggesting that this PTM might affect Akt1 recruitment to subcellular compartments. For some proteins, such as Hck, depalmitoylation leads to their accumulation in lysosomes (Sato et al., 2009). Thus, we wondered if the Akt1-C344S puncta represented localization to these organelles. Indeed, Akt1 puncta colocalized with LysoTracker red, a live staining dye that accumulates in low pH environments such as the lumen of lysosomes (Figure 4B and Supplementary Figure 1). Importantly, cells transfected with WT Akt1-YFP and treated with the palmitoylation inhibitor 2-bromopalmitate (2-BP), also exhibited this punctuated pattern, in around 30% of the cells (Figure 4C), arguing that lysosomal localization was due to the missing PTM and not merely due to a structural effect of the mutation of a cysteine 344 to serine, unrelated to the lipid addition. These results suggested that Akt1 might play a cellular function in lysosomes and that depalmitoylation facilitates, or be required for, lysosome targeting. Surprisingly, when we co-transfected WT Akt1-CFP and Akt1-C344S-YFP, we observed WT Akt in puncta, where Akt1-C344S was also present (Figure 4D), suggesting that recruitment of WT Akt1 to lysosomes could be induced by Akt1-C344S itself. Given that only a small percentage of cells show this localization, our data also suggests that other factors are necessary for Akt1 to reach this organelle.

To find a potential role of Akt1 in lysosomes, we used the STRING database (Szklarczyk et al., 2017) to analyze the list of already known Akt substrates (Moritz et al., 2010; Hornbeck et al., 2015). We then performed a gene ontology (GO) analysis of the biological processes linked to Akt targets associated to lysosomes and other connected organelles, and found an enrichment in GO terms related to the response to reactive oxygen species, as well as to autophagy and apoptosis (Supplementary Figure 2). Consistently, we found a total of 24 already known Akt substrates that are associated with the regulation of autophagy (Supplementary Figure 3). This analysis is in good agreement with recent reports demonstrating that Akt regulates lysosome-dependent autophagy (Wang et al., 2012; Matsuda-Lennikov et al., 2014; Arias et al., 2015).

The above analysis suggested that an autophagy-inducing input, such as oxidative stress, might lead to Akt relocalization to lysosomes. To test that possibility, we transfected HeLa Kyoto cells with WT Akt1-YFP and stimulated cells with two well-known inducers of oxidative stress: hydrogen peroxide (H_2_O_2_) (Chen et al., 2008) and photodamage (Kessel and Arroyo, 2007). Notably, both stimuli caused a significant proportion of cells with WT Akt1 to form cytoplasmic puncta (Figures 4E,F and Supplementary Figure 1B). The effect was much larger when we repeated this experiment using the palmitoylation deficient Akt1, resulting in approximately 50% and 75% of cells to show a punctuated pattern that colocalized with lysosomes in response to H_2_O_2_ and photodamage, respectively (Figures 4E,F and Supplementary Figure 1C). These results support the notion that depalmitoylated Akt1 has a larger propensity to be recruited to lysosomes in response to inducers of oxidative stress and autophagy, and suggest that regulated depalmitoylation might be a normal step required for lysosome targeting.

Overall, these results indicated that impairing Akt S-palmitoylation does not prevent Akt translocation to the PM but increases Akt recruitment to lysosomes, a process stimulated by inducers of oxidative stress and autophagy.

### Akt Recruitment to Lysosomes Is Accompanied by Golgi Disassembly and Nuclear Condensation

**FIGURE 5:**
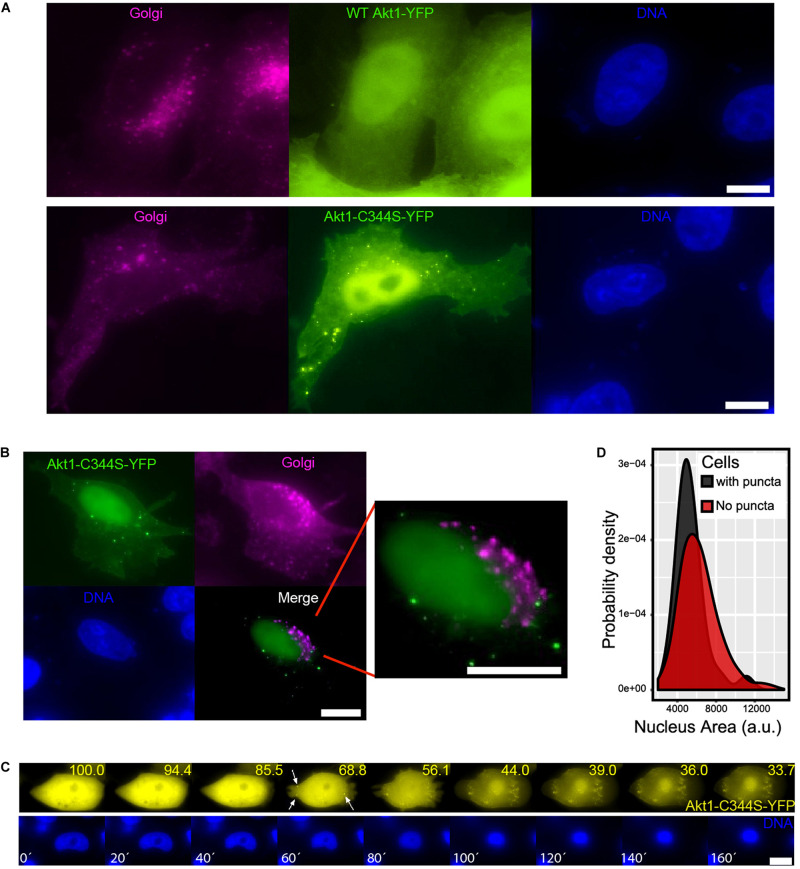
Akt recruitment to lysosomes is accompanied by Golgi disassembly and nuclear condensation. **(A)** HeLa Kyoto cells were co-transfected with a trans-Golgi fluorescent marker (TGN38-FRB-CFP, magenta) and either WT-YFP or Akt1-C344S-YFP (yellow). Dissociation of the typical Golgi structure into vesicles can be seen in cells displaying Akt puncta. DAPI was used to reveal cell nuclei (blue); scale bar, 5 μm. **(B)** Akt puncta (green) do not colocalize with Golgi (magenta). **(C)** Puncta formation in Akt1-C344S cells is accompanied by cell blebbing, nuclear condensation and fluorescence loss (yellow numbers indicate the percentage of yellow fluorescence remaining in the cell at each time point, compared to *t* = 0). HeLa Kyoto cells constitutively expressing H2B-mCherry (blue) were transfected with a plasmid coding for Akt1-C344S-YFP (yellow). Live cells were imaged every 20 min. Every time point (minutes, white numbers), we acquired a z-stack with 3 z-steps of 1 μm in 2 different wavelengths (RFP, exposure: 0.2 s and YFP, exposure: 0.5 s). Bar, 10 μm. **(D)** Akt1-C344S cells displaying Akt puncta show reduced nuclear area compared to WT Akt1-YFP expressing cells (*p* < 0.01 according to a Student’s *t*-test).

Interestingly, virtually all the cells displaying Akt in lysosomes also exhibited a change in Golgi structure from the typical perinuclear network to dispersed vesicles (Figure 5A). As expected, these vesicles do not colocalize with Akt puncta (Figure 5B). The change in Golgi structure is reminiscent of that observed during cell death (Foiani and Bartek, 2014). Photodamage induced by live-imaging microscopy revealed that Akt localization to lysosomes appeared during a process which also included nuclear condensation and cell blebbing (Figure 5C and Supplementary Movies 1 and 2). Consistently, unstimulated cells displaying Akt1-C344S in lysosomes presented reduced nuclear area compared with normal cells expressing WT Akt1 (Figure 5D).

### Palmitoylation-Deficient Akt Blocks Cell Differentiation

**FIGURE 6:**
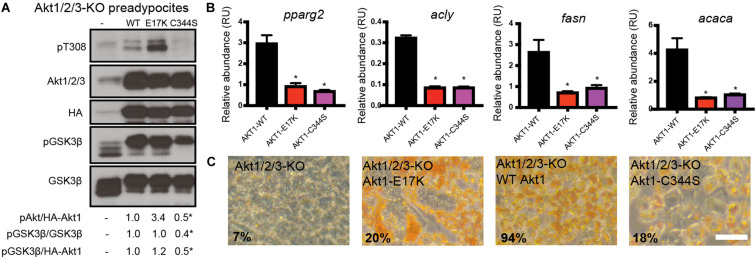
Akt1-C344S cannot support pre-adipocyte differentiation. **(A)** UBC-Cre Akt1/2/3 KO brown pre-adipocytes were infected with pBABE-pura-HA retroviruses into which WT Akt1, Akt1-E17K or Akt1-C344S coding sequences were cloned. Protein extracts were analyzed by western blot using the indicated antibodies. Numbers below correspond to the ratios of phospho-T308 Akt (pAkt) to total HA-tagged Akt, phospho-GSK3β (pGSK3β) to total GSK3β and pGSK3β to total HA-tagged Akt. Differences in Akt T308 and GSK3β phosphorylation between cells expressing WT HA-Akt1 and HA-Akt1-C344S were significant (ANOVA). **(B)** RT-qPCR analysis shows the expression of genes normally induced during *de novo* lipogenesis and adipocyte differentiation in cells expressing different Akt versions. Tbp expression was used as a housekeeping gene in all RT-PCR experiments. *p < 0.05 compared to WT Akt1 (ANOVA). **(C)** Oil Red O (ORO) staining reveals lipid accumulation in cells expressing different Akt versions (scale bar, 30 μm). The numbers show the area percentage in red. Cells expressing each of the Akt1 mutants display significant less amount of lipid accumulation compared to cells expressing WT Akt1 (ANOVA).

Autophagy is highly active during differentiation (Mizushima and Levine, 2010), particularly in the adipose tissue (Singh et al., 2009). Therefore, we wondered whether cells expressing the S-palmitoylation mutant Akt1-C344S have alterations in this Akt-dependent differentiation process (Cederquist et al., 2017). To assess this, we used an *in vitro* Akt-dependent adipogenic differentiation model, in which preadipocytes are induced to differentiate using a standard 10-day protocol. To test the role of Akt, we used a brown preadipocyte cell line in which the triple knock-out (KO) of Akt1/2/3 may be induced, allowing us to express our WT or C344S mutants as the sole source of Akt (see section “Materials and Methods”). As with HeLa Kyoto cells, we observed that Akt1-C344S displays significantly diminished T308 phosphorylation in these triple Akt KO cells before initiating the differentiation protocol (Figure 6A). Remarkably, we found that Akt1-C344S was functionally defective, as determined by a clear reduction in the phosphorylation levels of GSK3β, a canonical Akt substrate (Figure 6A). Finally, we found that neither Akt1-C344S nor Akt1-E17K, which stimulates cell proliferation, could replace WT Akt1 in its ability to support preadipocyte differentiation, as determined by expression analysis of key genes related to de novo lipogenesis and adipocyte differentiation as well as the ability to accumulate lipids -Oil Red O (ORO) staining- (Figures 6B,C).

## Discussion

A growing body of evidence supports the idea that recruitment of components of the Akt pathway to particular subcellular compartments may be central to determine the specific set of Akt substrates targeted and therefore the particular group of Akt functions activated (Menon et al., 2014; Marat et al., 2017). During the last few years, novel regulatory mechanisms of Akt activity were described, such as phosphorylations (Liu et al., 2014), ubiquitination (Yang et al., 2009), proline hydroxylation (Guo et al., 2016) and methylation (Yoshioka et al., 2016; Guo et al., 2019; Wang et al., 2019). Quite some work still needs to be done to understand the way in which the activity and localization patterns of this kinase is controlled. For example, it was recently shown that production of PI34P2 may account for isoform- and site- specific activation of Akt (Liu et al., 2018).

Here, we present evidence for a new Akt PTM: S-palmitoylation. This reversible modification has been shown to increase protein surface hydrophobicity and membrane affinity, modulating protein sorting and stability (Linder and Deschenes, 2007), phosphorylation and activity (Salaun et al., 2010), as well as numerous cellular processes, including apoptosis (Chakrabandhu et al., 2007). Interestingly, not all the algorithms predicted an Akt S-palmitoylation consensus as CSS-Palm. While SwissPalm (Blanc et al., 2015) predicts the same consensus as CSS-Palm, SeqPalm (Li et al., 2015) estimates a score for C344 palmitoylation which is not high enough to be considered as putative consensus. Our experimental data, however, is consistent with CSS-Palm and SwissPalm predictions, implying that prediction accuracy may vary depending on the protein analyzed. The Akt S-palmitoylation consensus seems to be highly conserved across evolution, and thus we are confident that this PTM plays an important role in other species. Our findings showing that Akt is palmitoylated at C344 in mammalian cells could be of paramount importance to understand Akt partitioning throughout internal cell membranes. S-palmitoylation was reported for some signaling molecules of the Akt pathway, such as Ras (Magee et al., 1987) and eNOS (Haynes et al., 2000). In this line of evidence, it was recently described that a novel Akt phosphatase, Small CTD Phosphatase 1 (SCP1) depends on palmitoylation at Golgi membranes in order to shuttle to the PM, where it would inhibit Akt activity (Liao et al., 2017). Most probably, Akt palmitoylation takes place after or during Akt binding to internal membranes, a process increasingly studied in the last few years (Siess and Leonard, 2019; Sugiyama et al., 2019; Yudushkin, 2019). Our results revealing the presence of Akt and PDK1 in Golgi membranes of unstimulated HeLa Kyoto cells are compatible with basal Akt palmitoylation by PATs residing on these membranes. Once palmitoylated, it is likely that these modified Akt molecules shuttle between different internal membranes as it has been shown for other palmitoylated proteins (Sato et al., 2009). Unless associated to a lipid binding protein (such as a G-protein GDP-dissociation inhibitor (GDI)) that could isolate the palmitate, the presence of soluble palmitoylated Akt molecules is unlikely (Olofsson, 1999). Further experiments are needed to precisely understand where and how is Akt palmitoylated as well as which is/are the PAT(s) responsible for this PTM and which are the stimuli that regulate it.

Our results showed that a mutant Akt1 that cannot undergo S-palmitoylation has reduced phosphorylation levels. A straightforward explanation for this effect is that the unpalmitoylated Akt1 fails to associate correctly with the membrane where it is modified by PDK1. However, molecular dynamics simulations of Akt1 with different PTMs suggested that cysteine 344 and threonine 308 are structurally closely connected, since modifications of C344 cause a clear change in the behavior/solvent exposure of T308, and vice versa. Thus, Akt S-palmitoylation might produce an allosteric effect modifying the neighborhood of T308, in a way that could conceivably affect the ability of PDK1 to act on this site. In summary, S-palmitoylation might affect the exact positioning, and thus the exposure, of this phosphorylation target amino acid. Moreover, palmitoylation and phosphorylation might combine synergistically to alter Akt1 function: one of the functions attributed to T450 phosphorylation is to reduce Akt ubiquitination, affecting Akt conformation and stability (Risso et al., 2015). Consistently, S-palmitoylation has been shown to modulate protein ubiquitination (Linder and Deschenes, 2007). Since Akt can be ubiquitinated at K8, 14 and 284, these results raise the possibility that S-palmitoylation plays a role not only in regulating Akt activation status through phosphorylation but also indirectly through Akt ubiquitination. Further experiments will be necessary to address this statement.

Akt has been described as a Phafin2 and Beclin 1 kinase, regulating lysosome-dependent autophagy (Wang et al., 2012; Matsuda-Lennikov et al., 2014; Arias et al., 2015). Recently, the existence of pools of AKT that are sensitive to lysosome positioning have been demonstrated (Jia and Bonifacino, 2019). We found that the Akt1-C344S mutation induces Akt relocalization to cytoplasmic puncta that correspond to lysosomes, a process stimulated by oxidative stress and linked to autophagy. The palmitoylation inhibitor 2-BP elicited the same relocalization pattern in WT Akt1, confirming these results. Moreover, blocking Akt1 S-palmitoylation impaired the ability of preadipocytes to accumulate lipids and to differentiate, a process also associated to cell autophagy (Singh et al., 2009; Mizushima and Levine, 2010). This is particularly relevant, since the adipose tissue largely depends on the existence of a proper balance between lipid storage and utilization, a process basically mediated by insulin signaling and the Akt pathway (Cederquist et al., 2017). The observation that the oncogenic Akt1-E17K mutant, which induces cell proliferation and survival, also impaired the ability of preadipocytes to accumulate lipids and in general to differentiate further supports the idea of a complex Akt molecular code in which different PTMs could trigger higher or lower Akt activity depending on the molecular function, subcellular compartment or biological process regulated by this kinase. Moreover, E17K mutation elicits different phenotypes depending on the isoform of Akt affected (Manning and Toker, 2017). Our conclusions regarding the function of Akt S-palmitoylation are based on experiments with a palmitoylation-deficient Akt mutant and the use of 2-BP, a compound that blocks general palmitoylation. While C344S mutation could affect the structure and function of Akt independently of Akt S-palmitoylation, 2-BP, for its part, could have non-specific effects by affecting palmitoylation of other proteins in addition to Akt. However, the fact that two different experimental strategies generate similar effects suggests that the results are genuinely associated with the role of Akt S-palmitoylation. Future experiments should complement this approach by analyzing the phosphorylation and specific subcellular localization of palmitoylated and non-palmitoylated WT Akt. Further research should also address whether PIP3 binding play any role in the relocalization of unpalmitoylated Akt, for example by studying the localization of a ΔPH-Akt1-C344S double mutant.

Understanding the interplay between the patterns of PTMs and subcellular localization of a given signaling protein not only represents a progress in the understanding of the mechanisms governing the regulation of the studied protein, but also a way to understand general mechanisms by which cells integrate, translate and transduce molecular codes into spatial information cues and vice versa. In the case of Akt, a protein involved in tumor progression (Altomare and Testa, 2005; Riggio et al., 2012) and in viral infections, including coronavirus (Tsoi et al., 2014), fine-tuning of these biological processes and functions can be essential for cell decision-making mechanisms during disease onset and progression. While we were able to observe Akt palmitoylation in unstimulated cells, how stimulation affects Akt palmitoylation remains an open question. Further experiments will be needed in order to understand the detailed mechanisms that govern Akt S-palmitoylation, localization and function and which stimuli regulate this PTM. However, our results leave us a step closer to deciphering the Akt molecular code. Particularly, our data are compatible with a role of Akt S-palmitoylation in phosphorylation, localization and function of this kinase, influencing key processes like autophagy, cell differentiation and cell death.

## Data Availability Statement

The raw data supporting the conclusions of this article will be made available by the authors, without undue reservation.

## Author Contributions

MB and AC-L designed the research and wrote the manuscript. MB performed most of the experiments. MB, EP, CMC, AV, and AA performed microscopy and image analysis. MVM and MMC performed ABE assays. LA and FGvdG designed and performed 3H-palmitate labeling experiments. AZ performed MD simulations. CMC and DAG designed and performed adipocyte differentiation experiments. MB, CMC, AV, AA, LA, and MVM performed western blot experiments. MV assisted in cell culture experiments. All authors contributed to the article and approved the submitted version.

## Conflict of Interest

The authors declare that the research was conducted in the absence of any commercial or financial relationships that could be construed as a potential conflict of interest.

## Supplementary Material

Supplementary Figure 1 Akt1-C344S cytosolic puncta colocalize with lysosomes. **(A)** HeLa Kyoto cells were transfected with a plasmid coding for Akt1-C344S-YFP (green) and live stained with Lysotracker Red to observe lysosomes (magenta); scale bar, 5 μm. 3D Surface Plots of specific cytosolic areas are shown. **(B)** HeLa Kyoto cells were transfected with a plasmid coding for Akt1-C344S-YFP. Oxidative stress was induced by photo-damage as in Figure 4F (left) or by treatment with 10 mM hydrogen peroxide for 3 h as in Figure 4E (right). Arrows indicate cells displaying Akt in puncta. Scale bar, 5 μm. **(C)** HeLa Kyoto cells were transfected with a plasmid coding for WT Akt1-YFP. Cytoplasmic Akt puncta in cells with 10 mM hydrogen peroxide (3 h) also colocalize with lysosomal structures. Scale bar, 5 μm.

Supplementary Figure 2 Akt substrates and functions in ERES, Golgi and lysosome membranes. **(A,C,E)** The protein network displays ER- **(A)**, Golgi- **(C)** and lysosomes- **(E)** associated Akt substrates and their interactions, respectively (according to GO annotation). These networks were obtained using the STRING database (all interaction sources), requiring at least an intermediate level of confidence (>0.400). Line thickness indicates the strength of data support while physical distances between two nodes along an edge in a graph has no meaning. Colored nodes: query proteins; Empty nodes: proteins of unknown 3D structure; Filled nodes: some 3D structure is known or predicted. **(B,D,F)** GO analysis of ER- **(B)**, Golgi- **(D)** and lysosomes- **(F)** associated Akt substrates. Plots show the top ten Biological Processes GO terms with significant enrichment. Too general, non-informative or repetitive terms were not included. False Discovery Rate (the expected proportion of erroneously rejected null hypotheses among all rejected ones, where each null hypothesis is related to the association of a given gene with a particular GO term) and Observed Protein Count (numbers next to each bar) for each term are shown. The False Discovery Rate bars are presented in log-10 scale. GO categories shown are not mutually exclusive.

Supplementary Figure 3 Interaction network of Akt substrates which were GO annotated as related to regulation of autophagy. This network was obtained using the STRING database (all interaction sources), requiring at least an intermediate level of confidence for interactions. Line thickness indicates the strength of data support while physical distances between two nodes along an edge in a graph has no meaning. Colored nodes: query proteins; Empty nodes: proteins of unknown 3D structure; Filled nodes: some 3D structure is known or predicted.

Supplementary Table 1 Predicted S-palmitoylation motifs in different Akt1 homologs. CSS-Palm software was used with a high stringency threshold. CSS-Palm 4.0 includes a fourth-generation Group-based Prediction System algorithm and the latest training data set, containing 583 palmitoylation sites from 277 distinct proteins.

Supplementary Movies 1, 2 *In vivo* imaging of Akt1 puncta formation. *In vivo* imaging of HeLa Kyoto cells constitutively expressing H2B-mCherry (blue) and transfected with a plasmid coding for Akt1-C344S-YFP (yellow). Cells were imaged every 20 min. Every time point, a Z Series was performed with 3 z steps of 1 μm for 2 different wavelengths (RFP, exposure: 0.2 s; YFP, exposure: 0.5 s). Puncta formation in Akt1-C344S-YFP overexpressing cells is accompanied by nuclear condensation, cell blebbing and fluorescence loss.
