# Supplementary figures and images for: Akt Is S-Palmitoylated: A New Layer of Regulation for Akt
Source: Front Cell Dev Biol. 2021 Feb 15;9:626404. doi: 10.3389/fcell.2021.626404 (PMC7917195; doi:10.3389/fcell.2021.626404)

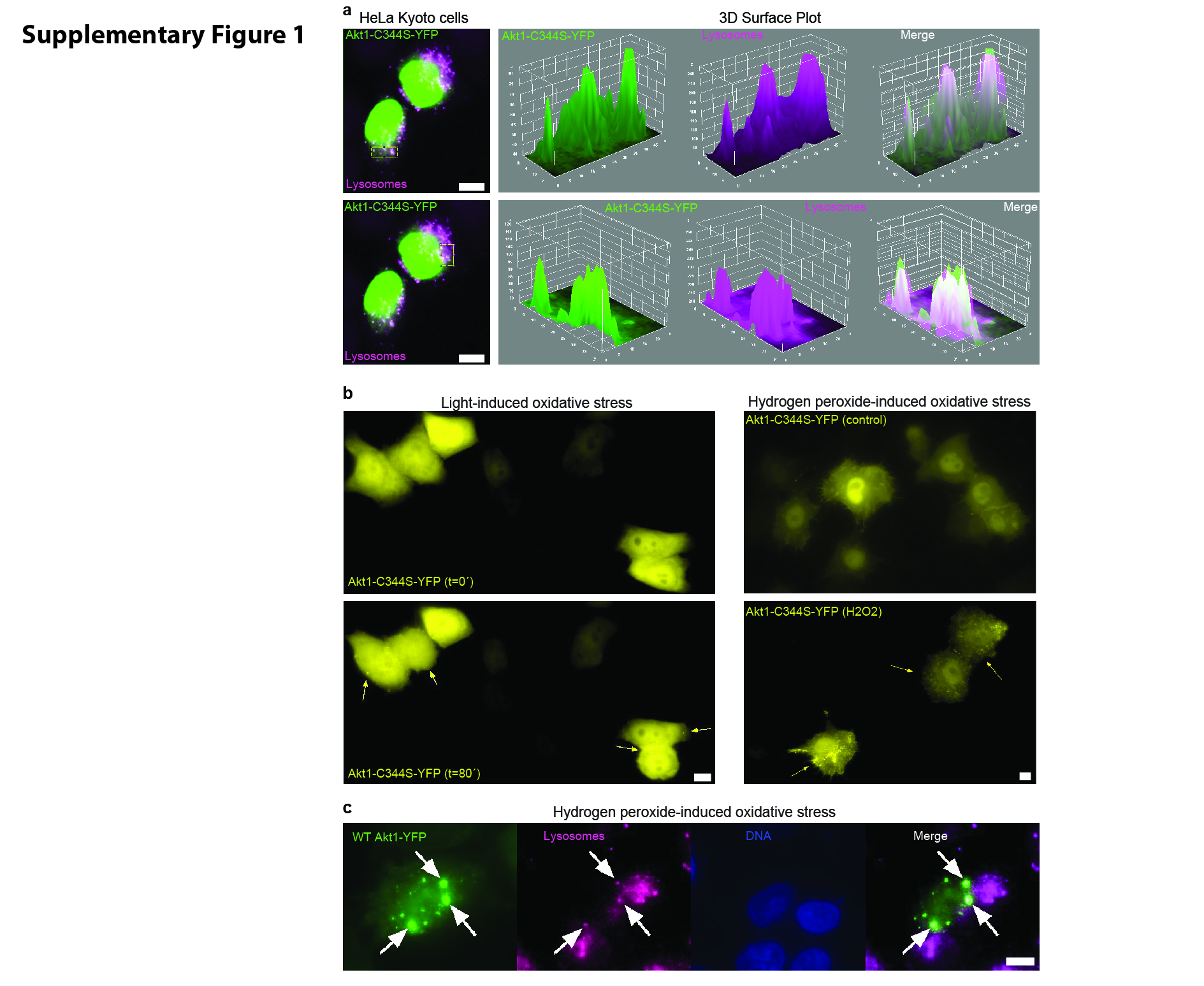

Supplement: Supplementary Figure 1 — Akt1-C344S cytosolic puncta colocalize with lysosomes. (A) HeLa Kyoto cells were transfected with a plasmid coding for Akt1-C344S-YFP (green) and live stained with Lysotracker Red to observe lysosomes (magenta); scale bar, 5 μm. 3D Surface Plots of specific cytosolic areas are shown. (B) HeLa Kyoto cells were transfected with a plasmid coding for Akt1-C344S-YFP. Oxidative stress was induced by photo-damage as in Figure 4F (left) or by treatment with 10 mM hydrogen peroxide for 3 h as in Figure 4E (right). Arrows indicate cells displaying Akt in puncta. Scale bar, 5 μm. (C) HeLa Kyoto cells were transfected with a plasmid coding for WT Akt1-YFP. Cytoplasmic Akt puncta in cells with 10 mM hydrogen peroxide (3 h) also colocalize with lysosomal structures. Scale bar, 5 μm. [file Image_1.JPEG]

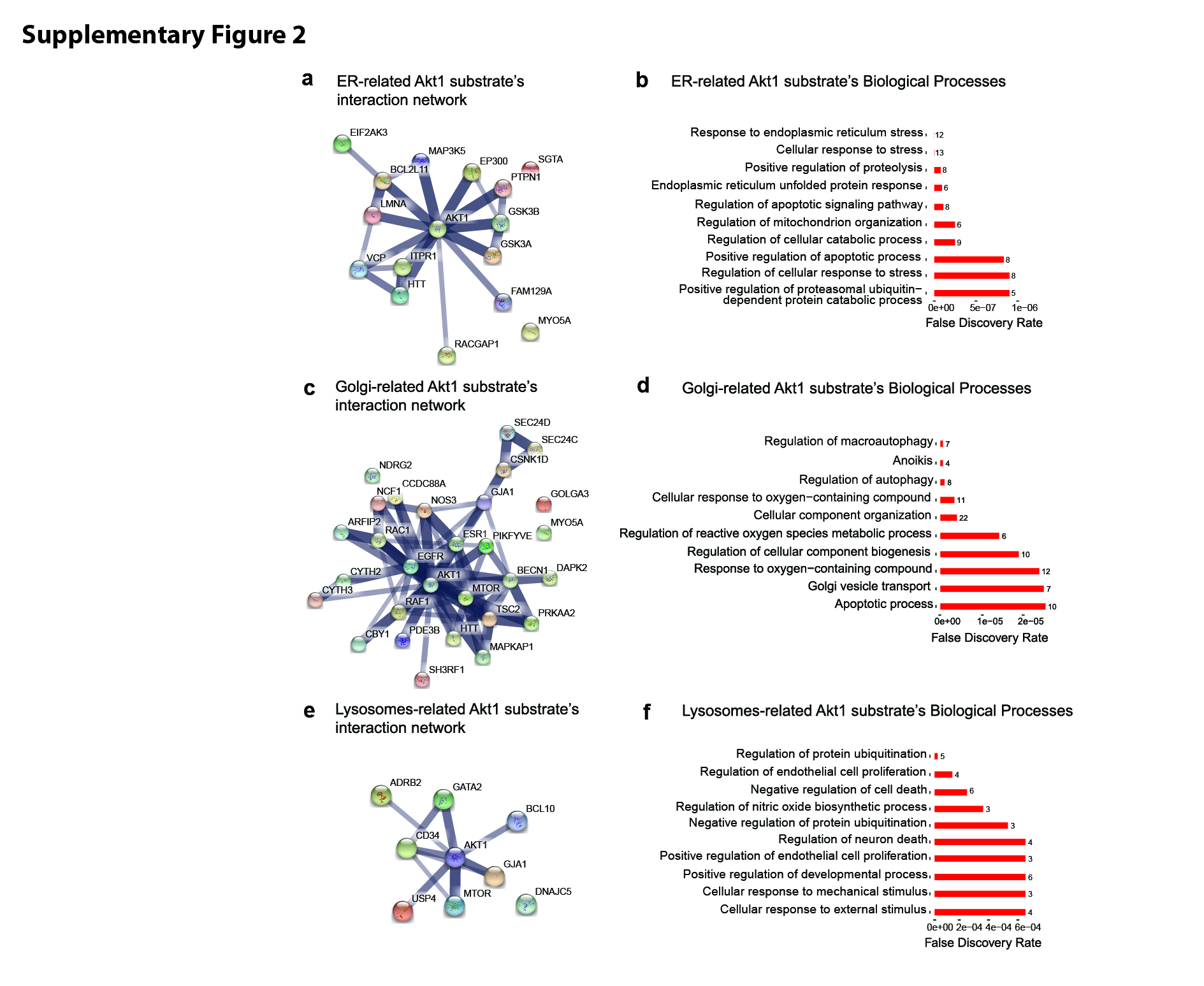

Supplement: Supplementary Figure 2 — Akt substrates and functions in ERES, Golgi and lysosome membranes. (A,C,E) The protein network displays ER- (A), Golgi- (C) and lysosomes- (E) associated Akt substrates and their interactions, respectively (according to GO annotation). These networks were obtained using the STRING database (all interaction sources), requiring at least an intermediate level of confidence (>0.400). Line thickness indicates the strength of data support while physical distances between two nodes along an edge in a graph has no meaning. Colored nodes: query proteins; Empty nodes: proteins of unknown 3D structure; Filled nodes: some 3D structure is known or predicted. (B,D,F) GO analysis of ER- (B), Golgi- (D) and lysosomes- (F) associated Akt substrates. Plots show the top ten Biological Processes GO terms with significant enrichment. Too general, non-informative or repetitive terms were not included. False Discovery Rate (the expected proportion of erroneously rejected null hypotheses among all rejected ones, where each null hypothesis is related to the association of a given gene with a particular GO term) and Observed Protein Count (numbers next to each bar) for each term are shown. The False Discovery Rate bars are presented in log-10 scale. GO categories shown are not mutually exclusive. [file Image_2.JPEG]

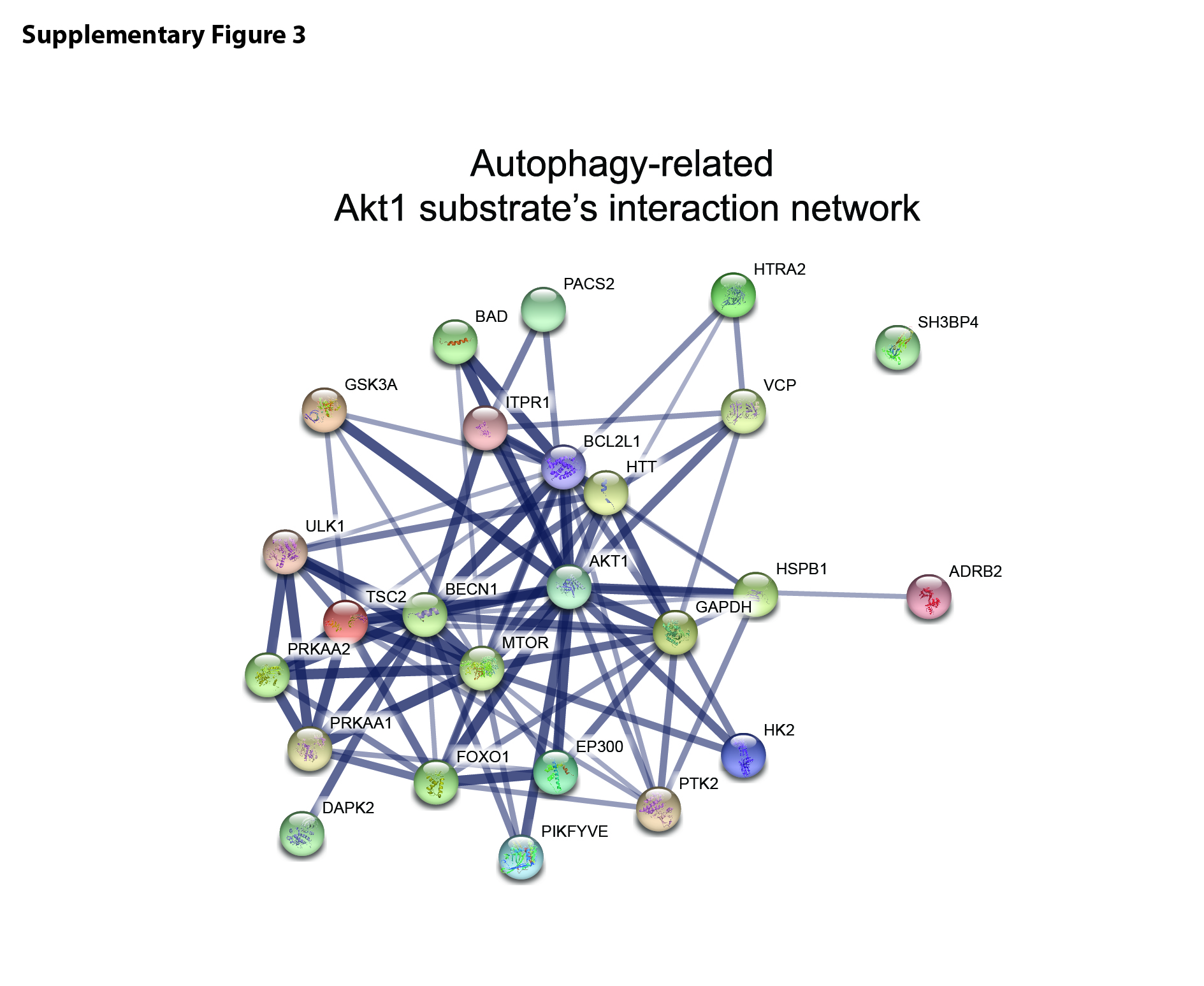

Supplement: Supplementary Figure 3 — Interaction network of Akt substrates which were GO annotated as related to regulation of autophagy. This network was obtained using the STRING database (all interaction sources), requiring at least an intermediate level of confidence for interactions. Line thickness indicates the strength of data support while physical distances between two nodes along an edge in a graph has no meaning. Colored nodes: query proteins; Empty nodes: proteins of unknown 3D structure; Filled nodes: some 3D structure is known or predicted. [file Image_3.JPEG]
