# Supplementary material for: Akt Is S-Palmitoylated: A New Layer of Regulation for Akt
Source: Front Cell Dev Biol. 2021 Feb 15;9:626404. doi: 10.3389/fcell.2021.626404 (PMC7917195; doi:10.3389/fcell.2021.626404)
Supplement: Supplementary Table 1 — Predicted S-palmitoylation motifs in different Akt1 homologs. CSS-Palm software was used with a high stringency threshold. CSS-Palm 4.0 includes a fourth-generation Group-based Prediction System algorithm and the latest training data set, containing 583 palmitoylation sites from 277 distinct proteins. [file Table_1.PDF]

SUPPLEMENTARY TABLE 1

Table S1. S-palmitoylation site predictions for different Akt homologs.

Species	ID	Position	Peptide	Score	Cutoff
H. sapiens	Akt1	344	VVMYEMMCGRLPFYN	4.941	4.222
H. sapiens	Akt2	345	VVMYEMMCGRLPFYN	4.941	4.222
H. sapiens	Akt3	341	VVMYEMMCGRLPFYN	4.941	4.222
R. norvegicus	Akt1	344	VVMYEMMCGRLPFYN	4.941	4.222
R. norvegicus	Akt2	345	VVMYEMMCGRLPFYN	4.941	4.222
R. norvegicus	Akt3	341	VVMYEMMCGRLPFYN	4.941	4.222
M. musculus	Akt1	344	VVMYEMMCGRLPFYN	4.941	4.222
M. musculus	Akt2	345	VVMYEMMCGRLPFYN	4.941	4.222
M. musculus	Akt3	341	VVMYEMMCGRLPFYN	4.941	4.222
C. elegans	Akt1	386	VVMYEMMCGRLPFYS	5.749	4.222
C. elegans	Akt2	373	VVMYEMMCGRLPFSA	5.133	4.222
S. pombe	Sck1	498	GVLVFEMCCGWSPFY	11.27	3.419
S. pombe	Sck1	499	VLVFEMCCGWSPFYA	5.849	4.222
S. cerevisiae	Sch9	606	GVLIFEMCCGWSPFF	13.117	3.419
S. cerevisiae	Sch9	607	VLIFEMCCGWSPFFA	4.995	4.222
